# Waiting-List and early posttransplant prognosis among ethnoracial groups: Data from the organ procurement and transplantation network

**DOI:** 10.3389/fsurg.2023.1045363

**Published:** 2023-01-30

**Authors:** Yangyang Wu, Kaikai Lv, Xiaowei Hao, Chao Lv, Wenhui Lai, Xinze Xia, Aibo Pang, Qing Yuan, Tao Song

**Affiliations:** ^1^ Department of Urology, The Third Medical Centre, Chinese People's Liberation Army (PLA) General Hospital, Beijing, China; ^2^ Graduate School, Medical School of Chinese People's Liberation Army (PLA), Beijing, China; ^3^ Graduate School, Hebei North University, Zhangjiakou, China; ^4^ Graduate School, Shanxi Medical University, Taiyuan, China

**Keywords:** kidney transplantation, waiting-list mortality, early posttransplant in-hospital mortality, kidney allocation system, primary nonfunction, racial/ethnic disparities

## Abstract

**Background:**

Racial/ethnic disparity in waiting-list mortality among candidates listed for kidney transplantation (KT) in the United States remains unclear. We aimed to assess racial/ethnic disparity in waiting-list prognosis among patients listed for KT in the United States in the current era.

**Methods:**

We compared waiting-list and early posttransplant in-hospital mortality or primary nonfunction (PNF) among adult (age ≥18 years) white, black, Hispanic, and Asian patients listed for only KT in the United States between July 1, 2004 and March 31, 2020.

**Results:**

Of the 516,451 participants, 45.6%, 29.8%, 17.5%, and 7.1% were white, black, Hispanic, and Asian, respectively. Mortality on the 3-year waiting list (including patients who were removed for deterioration) was 23.2%, 16.6%, 16.2%, and 13.8% in white, black, Hispanic, and Asian patients, respectively. The cumulative incidence of posttransplant in-hospital death or PNF after KT was 3.3%, 2.5%, 2.4%, and 2.2% in black, white, Hispanic, and Asian patients,respectively. White candidates had the highest mortality risk on the waiting list or of becoming too sick for a transplant, while black (adjusted hazard ratio, [95% confidence interval, CI], 0.67 [0.66–0.68]), Hispanic (0.59 [0.58–0.60]), and Asian (0.54 [0.52–0.55]) candidates had a lower risk. Black KT recipients (odds ratio, [95% CI] 1.29 [1.21–1.38]) had a higher risk of PNF or death before discharge than white patients. After controlling confounders, black recipients (0.99 [0.92–1.07]) had a similar higher risk of posttransplant in-hospital mortality or PNF as white patients than Hispanic and Asian counterparts.

**Conclusions:**

Despite having a better socioeconomic status and being allocated better kidneys, white patients had the worst prognosis during the waiting periods. Black recipients and white recipients have higher posttransplant in-hospital mortality or PNF.

## Introduction

Kidney transplantation (KT) is the best therapeutic option for the majority of patients with end-stage renal disease (ESRD) as it offers better quality of life and longer survival than other therapies (1, 2). Racial disparities are associated with lower socioeconomic status and longer transplant-waiting time (3, 4), which may affect the survival of patients on the waiting list and eventually contribute to worse graft and recipient survival in KT recipients (5, 6).

Previous studies have mainly focused on the racial disparities between whites and blacks in the long-term survival after transplantation of KT recipients; however, there is limited information on the racial disparities in the mortality rates of candidates awaiting KT. In the past 10 years, the proportion of Asian and Hispanic candidates in the United States has gradually increased, together with a decline in the proportion of white candidates (7). In the United States, the proportion of Asian and Hispanic patients on the waiting list increased from 7.5% and 17.7% in 2009 to 9.7% and 20.7% in 2019, respectively (7). However, only a few ethnoracially related KT studies have previously included Hispanic and Asian patients. Analysis of early posttransplant in-hospital mortality or primary nonfunction (PNF) may provide additional information on the prognosis of the waiting list, because sicker candidates tend to have a worse prognosis after KT. PNF, defined as failed function of the transplanted kidney, which necessitated continued maintenance dialysis (8) that occurred within 90 days post-KT, was included in the analysis.

The new Kidney Allocation System (KAS) implemented in 2014 increased the transplantation rates of highly sensitised individuals and reduced the known disparities in access to transplantation to some degree (9–11) whereas the relationship between KAS and waiting-list prognosis or early posttransplant prognosis remains unclear.

Kidney quality can be assessed using the Kidney Donor Profile Index (KDPI). Among kidney transplant recipients, a lower and higher KDPI are associated with longer and shorter predicted graft survival, respectively (12). Since the quality of the donated kidney can affect early posttransplant prognosis (12) we compared the differences in the changes in KDPI before and after KAS modification among races/ethnicities. The objectives of this study were to (a) compare baseline characteristics between white, black, Hispanic, and Asian patients listed for KT in the United States; (b) compare the waiting-list mortality* between these racial/ethnic groups; (c) compare early posttransplant in-hospital mortality or PNF between KT recipients across four groups; and (d) determine the role of KAS in the waiting list and early posttransplant prognoses.

## Materials and methods

### Study population

All adult patients (age ≥18 years) listed for only KT and no other organ transplants in the United States between July 1, 2004 and March 31, 2020 were identified in the Organ Procurement and Transplantation Network (OPTN) database, which includes data on all candidates awaiting KT in the United States. Individuals listed for multiorgan transplantation or removed on the day of enrollment (zero wait time) were excluded. The OPTN adult KT registration forms, which are completed by the transplantation centre clinicians, include details of the race/ethnicity of all recipients designated as white, black, Hispanic, or Asian. Due to their small number, patients belonging to other minorities were excluded from the study. The data reported here have been supplied by the UNOS as the contractor for OPTN. As the data was sourced from a public database, and study participants could not be identified directly or through linked identifiers, the study was exempted from ethics review.

### Study design and definitions

Demographic and clinical variables were recorded at the time of listing, determining the waiting-list prognosis, or at the time of transplant for ascertaining early posttransplant prognosis. Baseline characteristics and prognosis were compared between white, black, Hispanic, and Asian patients who were listed for KT during the study period. The primary endpoint (denoted as waiting-list death*) was a composite of death while on the waiting list or becoming too ill to undergo transplantation (removal from the waiting list owing to clinical deterioration). The cumulative incidence of these two events is denoted as cumulative waiting-list mortality*. Patients who underwent KT or were removed from the waiting list (for recovery or other reasons) were censored. The secondary endpoint (denoted as PNF^#^) was early posttransplant in-hospital death or PNF in patients who underwent KT. For analysis of waiting-list prognosis, patients were followed-up from the time of listing until death, KT, removal from the list, or the day of the last observation (March 31,2021). Patients who received KT were followed-up either until discharge, in-hospital death, posttransplant failure for up to 90 days, or the last day of the study period(March 31, 2021), whichever was earlier. To assess whether the relationship between race/ethnicity and patients' prognosis was related to centre volume, we divided the listing centres into three categories according to the distribution of patients enrolled at each centre during the study period as follows: low-volume (<50th percentile), medium-volume (50th–90th percentile), and high-volume (>90th percentile) centres (13). None of the participants had missing data for the following variables: age, sex, race/ethnicity, body mass index (BMI), blood type, dialysis, and dates of listing, transplant, death, or removal from the waiting list. Candidates with missing data for other variables were excluded from the multivariate analysis.

### Statistical analysis

Summary data are presented as the mean (standard deviation) or number (percentage). The differences between groups in baseline characteristics between ethnoracial groups were compared using the chi-square test or Student's *t*-test for categorical or continuous variables, respectively. The waiting list mortality* and PNF^#^ of participants are presented in the Kaplan–Meier curve and further compared using the log-rank test. A univariate Cox proportional hazards model was developed first. Subsequently, a multivariate Cox proportional hazards model was developed using backward stepwise selection, and all the variables in Table 1 were considered. A univariate logistic regression model was developed first, and a multivariate logistic regression model was then developed to evaluate racial/ethnic differences in PNF^#^. Segmental linear regression was used to ascertain the trend of waiting-list death* and PNF^#^ before and after KAS. The KDPI of kidneys allocated to patients before and after KAS implementation was compared using the *t*-test. The distribution of the causes of death among waiting-listed candidates and recipients were compared using the chi-square test.

**Table 1:** Baseline characteristics of white, black, Hispanic and Asian patients listed for a kidney transplantation.

	White (23,5568)	Black (15,3932)	Hispanic (90,152)	Asian (36,799)	*p*
Age, years (mean (SD))	53.73 (13.27)	50.32 (12.48)	49.26 (13.37)	52.17 (13.03)	<0.001
Sex, Male, (%)	14,7902 (62.8)	90,441 (58.8)	56,438 (62.6)	21,608 (58.7)	<0.001
ABO (%)					<0.001
A	94,948 (40.3)	38,894 (25.3)	26,178 (29.0)	9,221 (25.1)	
B	25,944 (11.0)	31,316 (20.3)	8,769 (9.7)	10,676 (29.0)	
O	105,634 (44.8)	77,312 (50.2)	53,298 (59.1)	14,393 (39.1)	
AB	9,042 (3.8)	6,410 (4.2)	1,907 (2.1)	2,509 (6.8)	
BMI (mean (SD))	28.66 (5.62)	29.43 (5.78)	28.36 (5.24)	25.69 (4.78)	<0.001
Obese (BMI ≥30)	90,818 (38.6)	67,327 (43.7)	31,878 (35.4)	6,503 (17.7)	<0.001
History of diabetes (%)	84,762 (36.0)	66,273 (43.1)	46,086 (51.1)	16,041 (43.6)	<0.001
Private insurance (%)	120,412 (51.1)	54,500 (35.4)	31,069 (34.5)	18,685 (50.8)	<0.001
CPRA ≥30 (%)	49,256 (20.9)	41,723 (27.1)	19,165 (21.3)	7,664 (20.8)	<0.001
Working for income (%)	90,175 (38.3)	43,185 (28.1)	22,749 (25.2)	13,778 (37.4)	<0.001
College or graduate degree (%)	132,627 (56.3)	75,727 (49.2)	25,848 (28.7)	22,231 (60.4)	<0.001
Dialysis before registration (%)	138,629 (58.8)	124,317 (80.8)	72,688 (80.6)	25,301 (68.8)	<0.001
Centre volume (%)					<0.001
Low volume	31,965 (13.6)	16,809 (10.9)	10,056 (11.2)	3,702 (10.1)	
Medium volume	124,893 (53.0)	83,771 (54.4)	45,330 (50.3)	16,493 (44.8)	
High volume	78,710 (33.4)	53,352 (34.7)	34,766 (38.6)	16,604 (45.1)	
Cause of ESRD (%)					<0.001
Glomerular diseases	37,514 (15.9)	16,366 (10.6)	10,639 (11.8)	7,630 (20.7)	
Hypertensive nephrosclerosis	34,586 (14.7)	52,636 (34.2)	16,164 (17.9)	6,908 (18.8)	
DM	62,208 (26.4)	48,878 (31.8)	39,604 (43.9)	12,715 (34.6)	
Other	101,260 (43.0)	36,052 (23.4)	23,745 (26.3)	9,546 (25.9)	

SD, standard deviation; BMI, body mass index (calculated as weight in kilograms divided by height in metres squared); CPRA, calculated panel reactive antibody; ESRD, end-stage renal disease; DM, diabetes mellitus.

Data were analysed using R (version 3.6.2). All statistical tests were two-sided, and *P *< 0.05 was used to define statistical significance.

## Results

**Figure 1:**
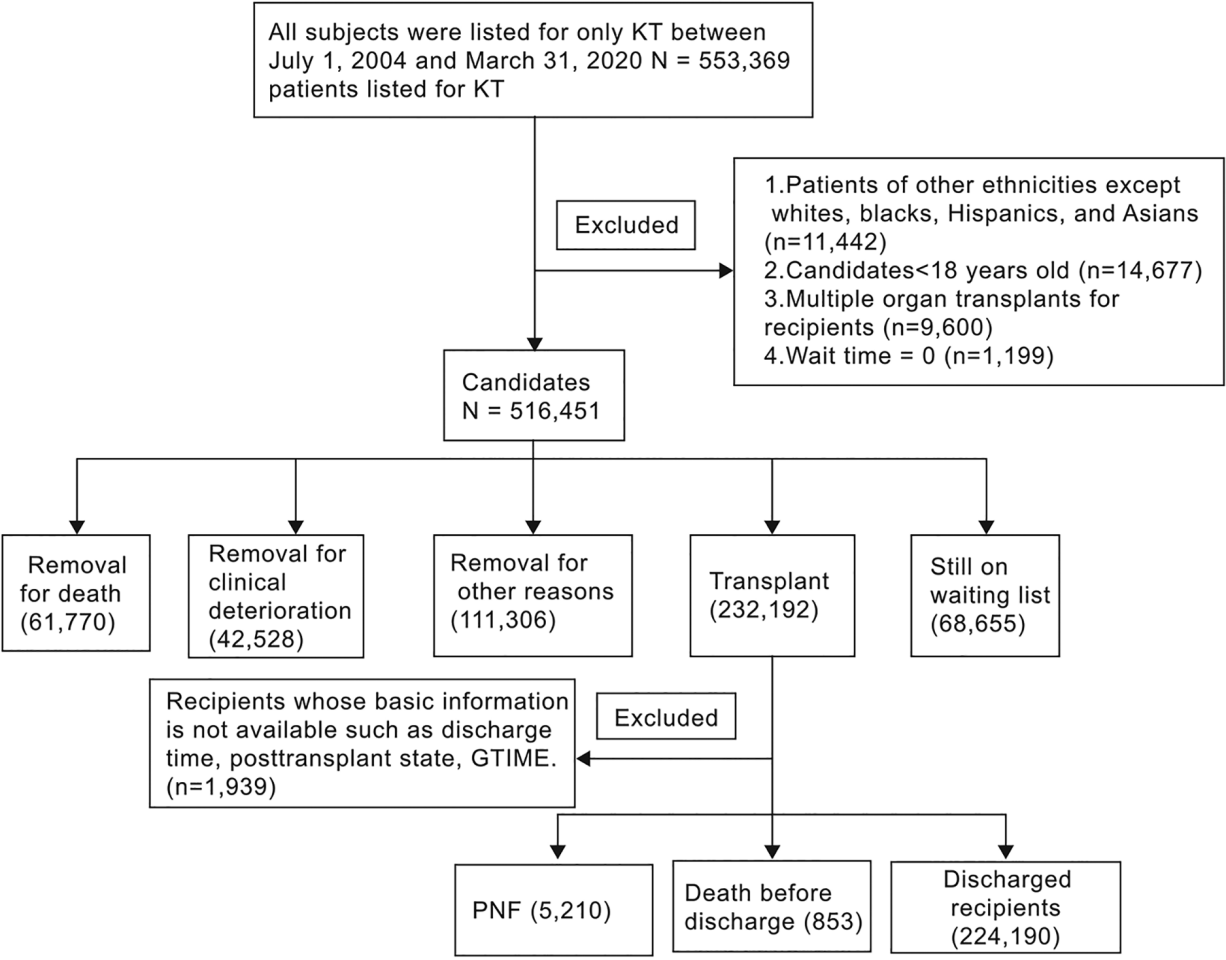
Flow diagram of the study participants. KT: kidney transplant; PNF: Primary nonfunction. Note: 1. GTIME, a variable derived from UNOS, indicates graft lifespan-kidney-days from transplant to failure/death/last follow-up; 2. Of the 853 deaths, 836 were simultaneously diagnosed with PNF.

During the study period, 553,369 patients in the United States were referred to transplant centres for only KT. Of these, 36,918 patients (6.7%) were excluded based on the predefined exclusion criteria (Figure 1). The remaining 516,451 patients were included in this analysis. Of these, 235,568 (45.6%) were white, 153,932 (29.8%) were black, 90,152 (17.5%) were Hispanic, and 36,799 (7.1%) were Asian (Figure 1).

Significantly (*P *< 0.001 for all), white patients were older than black, Hispanic, and Asian patients; were more likely to have type A blood (white 40.3% vs. black 25.3%, Hispanic 29.0%, and Asian 25.1%); have private insurance (51.1% vs. 35.4%, 34.5%, and 50.8%), and to work for income (38.3% vs. 28.1%, 25.2%, and 37.4%). Compared with black, Hispanic, and Asian patients, white patients were significantly (*P *< 0.001 for all) less likely to have a history of diabetes (white 36.0% vs. black 43.1%, Hispanic 51.1%, and Asian 43.6%) and dialysis before registration (58.8% vs. 80.8%, 80.6%, and 68.8%) or to register at a high-volume centre (33.4% vs. 34.7%, 38.6%, and 45.1%). White and Asian patients were less likely to have calculated panel-reactive antibody (CPRA) ≥ 30% than black and Hispanic patients (20.9% and 20.8% vs. 27.1% and 21.3%, respectively, *P *< 0.001). Black patients were more likely to be obese (43.7% vs. white 38.6%, Hispanic 35.4%, and Asian 17.7%) and were less likely to be diagnosed with glomerular diseases (10.6% vs. 15.9%, 11.8%, and 20.7%, *P *< 0.001), which is a cause of ESRD. Asian patients were more likely to have a college or graduate degree (60.4% vs. white 56.3%, black 49.2%, and Hispanic 28.7%, *P *< 0.001; Table 1).

The same trend was observed in transplant recipients, except for centre volume and CPRA. Black recipients were significantly less likely (*P *< 0.001) to receive the transplant at a high-volume centre (32.2% vs. white 34.5%, Hispanic 37.0%, and Asian 41.7%), and white recipients were significantly less likely (*P *< 0.001) to have CPRA ≥30% (21.0% vs. black 26.4%, Hispanic 23.3%, and Asian 22.3%). The donor age for white recipients was higher than that of black, Hispanic, and Asian recipients. White recipients were significantly less likely (*P *< 0.001) to have race/ethnicity-mismatched KT (14.6% vs. black 70.8%, Hispanic 56.2%, and Asian 77.7%), cold ischaemic time ≥12 h (41.2% vs. 61.9%, 53.1%, and 52.0%), human leukocyte antigen (HLA) mismatch ≥3 (41.2% vs. 91.0%, 80.5%, and 88.2%). White recipients were significantly more likely to access kidneys donated by living donors (43.9% vs. black 16.8%, Hispanic 30.4%, and Asian 27.6%, *P *< 0.001; Table 2).

**Table 2:** Clinical characteristics at time of transplantation in kidney transplant recipients and donors, by race/ethnicity.

	White (116,085)	Black (61,766)	Hispanic (37,518)	Asian (14,884)
**Recipient**				
Age, years (mean (SD))	53.29 (13.74)	51.13 (12.55)	48.91 (13.85)	52.12 (13.47)
Sex, Male, (%)	72,148 (62.2)	36,783 (59.6)	23,072 (61.5)	8,403 (56.5)
Obese (BMI ≥30)	41,043 (35.4)	25,073 (40.6)	11,873 (31.6)	2,177 (14.6)
History of diabetes (%)	33,081 (28.5)	22,585 (36.6)	14,822 (39.5)	4,997 (33.6)
Private insurance (%)	50,705 (43.7)	13,881 (22.5)	10,584 (28.2)	5,509 (37.0)
CPRA ≥30 (%)	24,383 (21.0)	16,284 (26.4)	8,752 (23.3)	3,317 (22.3)
Working for income (%)	44,039 (37.9)	15,564 (25.2)	9,580 (25.5)	5,021 (33.7)
College or graduate degree (%)	67,145 (57.8)	31,179 (50.5)	11,432 (30.5)	9,072 (61.0)
Dialysis before registration (%)	65,655 (56.6)	50,565 (81.9)	30,057 (80.1)	10,327 (69.4)
Center volume *N* (%)				
Low volume	13,447 (11.6)	7,175 (11.6)	4,542 (12.1)	1,510 (10.1)
Medium volume	62,580 (53.9)	34,720 (56.2)	19,093 (50.9)	7,170 (48.2)
High volume	40,058 (34.5)	19,871 (32.2)	13,883 (37.0)	6,204 (41.7)
Cause of ESRD (%)				
Glomerular diseases	23,076 (19.9)	8,303 (13.4)	6,260 (16.7)	4,105 (27.6)
Hypertensive nephrosclerosis	17,597 (15.2)	23,036 (37.3)	7,921 (21.1)	3,077 (20.7)
DM	24,701 (21.3)	17,252 (27.9)	12,834 (34.2)	3,964 (26.6)
**Donor**				
Age (mean (SD))	41.48 (14.54)	38.79 (14.95)	38.32 (14.87)	39.66 (16.46)
Obese (BMI ≥30)	31,208 (26.9)	19,276 (31.2)	11,005 (29.3)	3,641 (24.5)
Cold ischaemic time ≥12 h	47,835 (41.2)	38,264 (61.9)	19,921 (53.1)	7,739 (52.0)
Sex mismatch = Yes (%)	58,012 (50.0)	29,451 (47.7)	18,504 (49.3)	7,487 (50.3)
Race/ethnicity mismatch = Yes (%)	16,906 (14.6)	43,751 (70.8)	21,082 (56.2)	11,565 (77.7)
ABO incomparable = Yes (%)	1,157 (1.0)	825 (1.3)	350 (0.9)	323 (2.2)
HLA mismatch ≥3 (%)	89,335 (77.0)	56,219 (91.0)	30,185 (80.5)	13,130 (88.2)
Donor type = Living (%)	51,005 (43.9)	10,401 (16.8)	11,416 (30.4)	4,102 (27.6)
Deceased donor cause of death (%)				
Anoxia	22,560 (34.7)	18,118 (35.3)	8,939 (34.2)	3,926 (36.4)
Cerebrovascular/stroke	18,906 (29.1)	14,916 (29.0)	7,629 (29.2)	3,262 (30.3)
Head trauma	21,532 (33.1)	16,753 (32.6)	8,710 (33.4)	3,182 (29.5)

SD, standard deviation; BMI, body mass index (calculated as weight in kilograms divided by height in metres squared); CPRA, calculated panel reactive antibody; ESRD, end-stage renal disease; DM, diabetes mellitus; HLA, human leukocyte antigen.

### Waiting-List mortality

**Figure 2:**
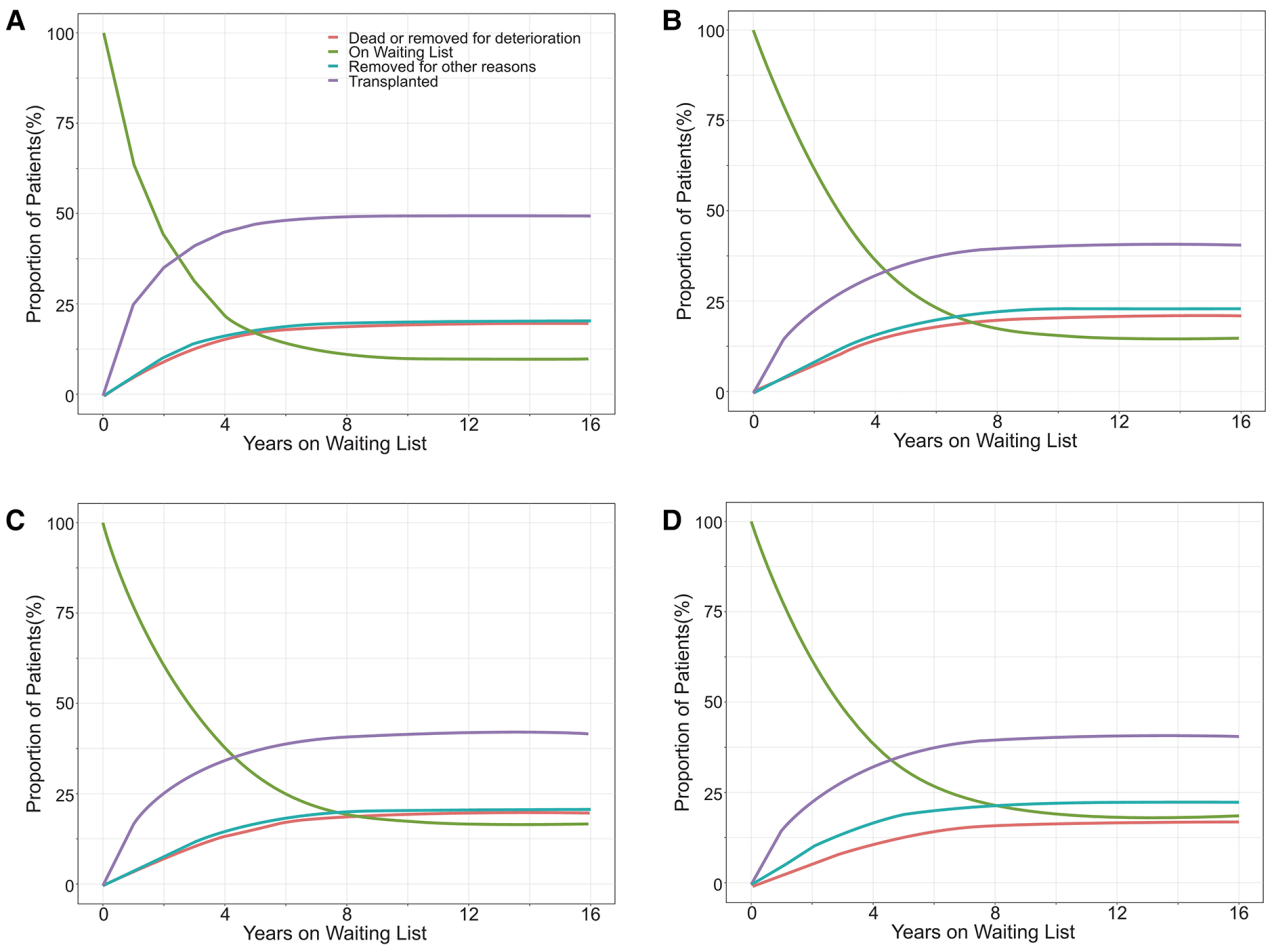
The proportion of outcomes for white (**A**), black (**B**), Hispanic (**C**), and Asian (**D**) patients listed for a kidney transplant in the United States.

Overall, 104,298 (20.2%) patients reached the primary end point (61,770 [12.0%] died on the waiting list and 42,528 [8.2%] were removed due to clinical deterioration) during the study period. Ultimately, 20.0%, 21.1%, 20.2%, and 17.7% of white, black, Hispanic, and Asian candidates, respectively, on the KT waiting list died or were removed due to clinical deterioration. Moreover, 49.6%, 40.5%, 42.0%, and 40.8% of white, black, Hispanic, and Asian candidates, respectively, received KT (Figure 2). The median [IQR] waiting time for a KT was 11.5 [4.4–26.7], 19.7 [6.4–40.8], 16.6 [5.4–38.4], and 19.8 [7.2–41.9] months for white, black, Hispanic, and Asian candidates, respectively.

**Figure 3:**
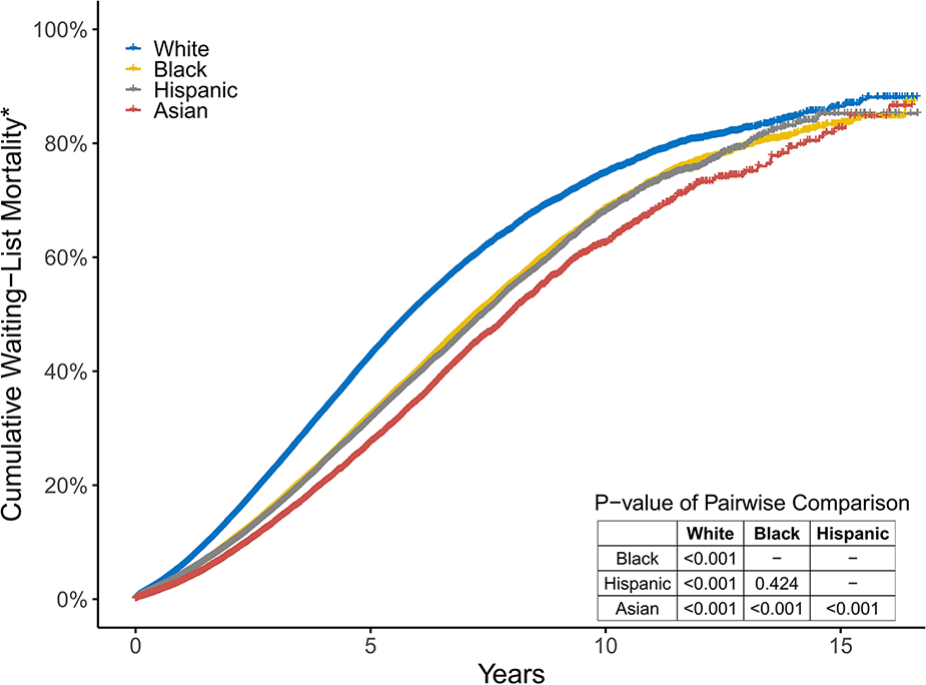
The cumulative percentage of those who died or became too sick for a transplant in the four groups. These two endpoints are simply referred to as Wait-List mortality*.

There was a significant difference in the cumulative waiting-list mortality* among the four groups. White patients had the highest risk of waiting-list mortality*, followed by black and Hispanic patients, while Asian patients had the lowest risk. The 3-year waiting-list mortality* of white, black, Hispanic, and Asian patients were 23.2%, 16.6%, 16.2%, and 13.8%, respectively (Figure 3).

In univariate Cox proportional hazards analysis, white candidates were at the highest risk of dying on the waiting list or becoming too sick for KT. After controlling for candidates' age, sex, blood type, BMI, history of diabetes, primary insurance, level of education, work state, dialysis time, CPRA, centre volume, causes of ESRD, and the implementation of KAS, white candidates still had the worst waiting-list prognosis, whereas black, Hispanic, and Asian candidates had a lower risk of poor prognosis (aHR [95% CI] 0.67 [0.66–0.68], 0.59 [0.58–0.60], and 0.54 [0.52–0.55]).

**Figure 4:**
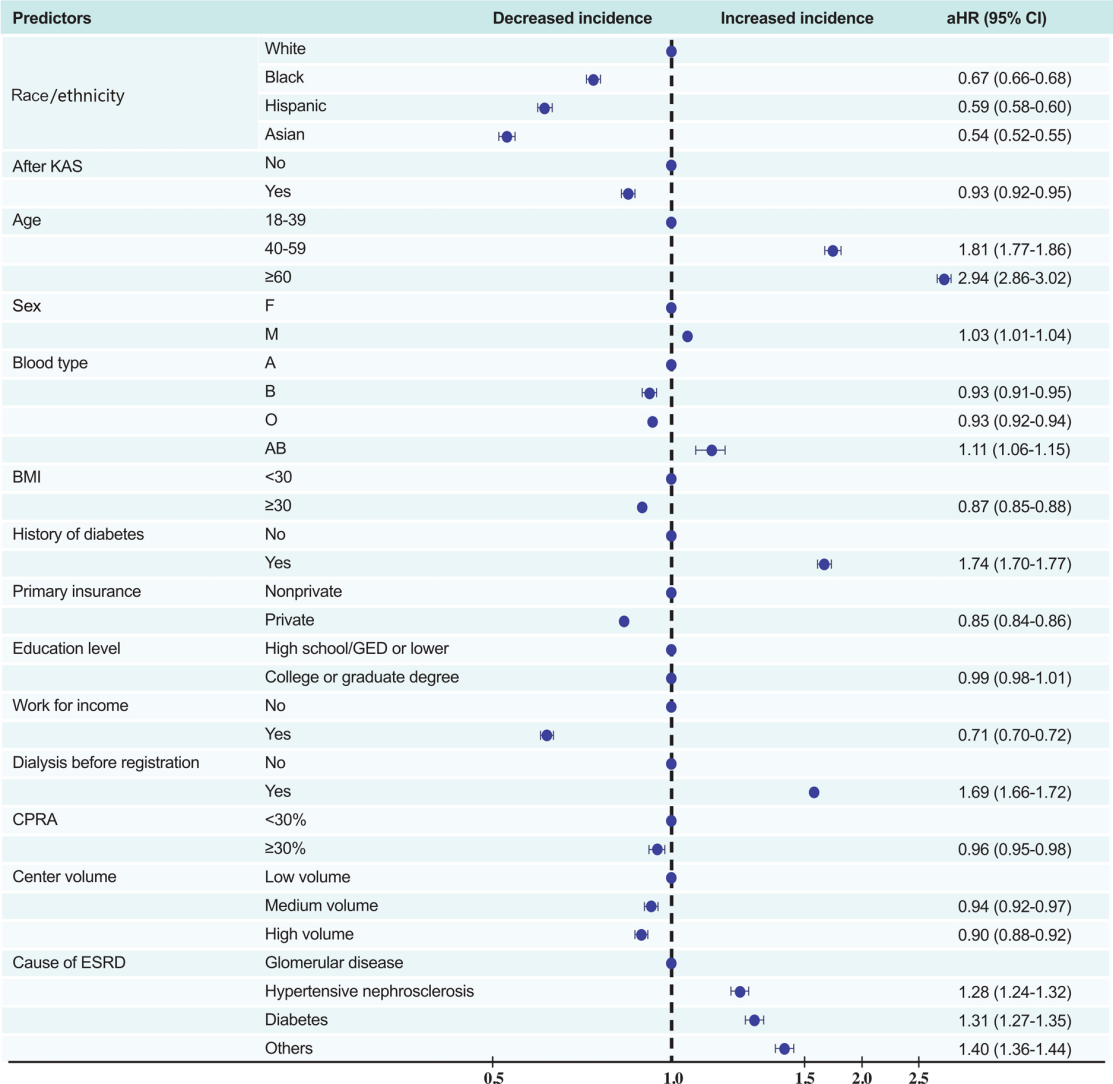
Multivariate predictors of waiting-list death or removal from the list owing to clinical deterioration. KAS, Kidney Allocation System BMI, body mass index (calculated as weight in kilograms divided by height in metres squared); CPRA, calculated panel reactive antibody; ESRD, end-stage renal disease; DM, diabetes mellitus.

Moreover, the implementation of KAS, BMI ≥30, possessing private insurance, and registration in a higher volume centre were associated with lower probability of waiting-list death*(aHR [95% CI] 0.93 [0.92–0.95], 0.87 [0.85–0.88], 0.85 [0.84–0.86], and 0.90 [0.88–0.92], respectively), whereas older age (patients ≥60 years), diabetes, and dialysis before KT registration were associated with higher probability of waiting-list death* (aHR [95% CI] 2.94 [2.86–3.02], 1.74 [1.70–1.77], and 1.69 [1.66–1.72], respectively). Employed patients were 29% less likely to die on the waiting list compared with unemployed patients (95% CI 0.70–0.72) (Figure 4).

### Posttransplant in-hospital mortality or PNF

**Figure 5:**
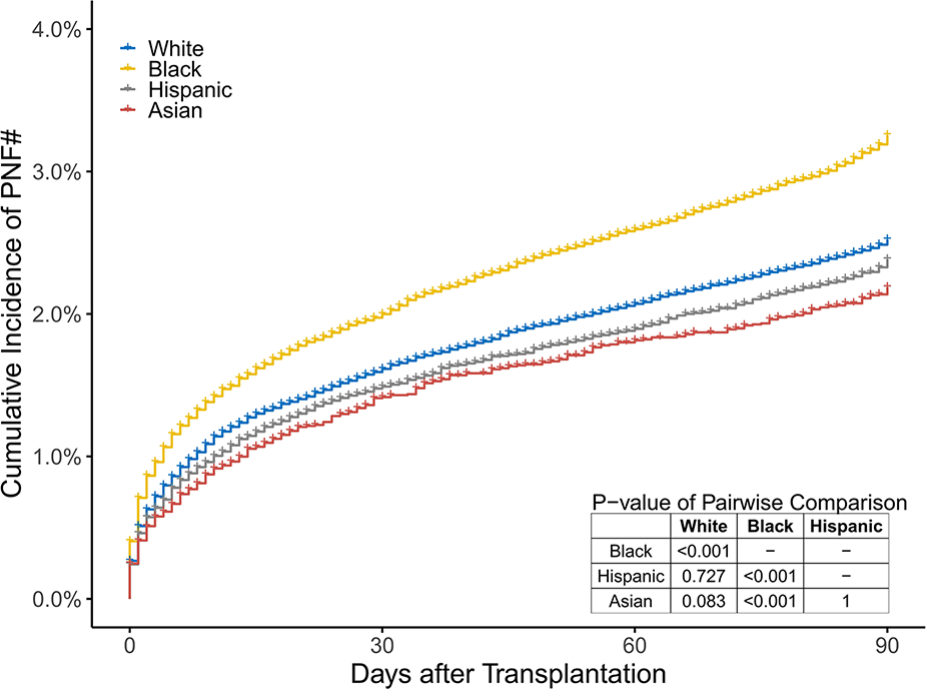
The cumulative percentage of recipients who died or suffered from PNF before discharge in the 4 groups. These two endpoints are simply referred to as PNF^#^.

Among 230,253 participants who received a KT and whose discharge status was known, 224,190 (97.4%) were discharged from the hospital without PNF; 5,210 (2.3%) recipients suffered from PNF before hospital discharge; and 853 (0.4%) died (including 836 recipients who were diagnosed with PNF before death) in the hospital. The cumulative incidence of PNF^#^ after KT was 3.3%, 2.5%, 2.4%, and 2.2% in black, white, Hispanic, and Asian recipients, respectively (*P *< 0.001; Figure 5).

**Figure 6:**
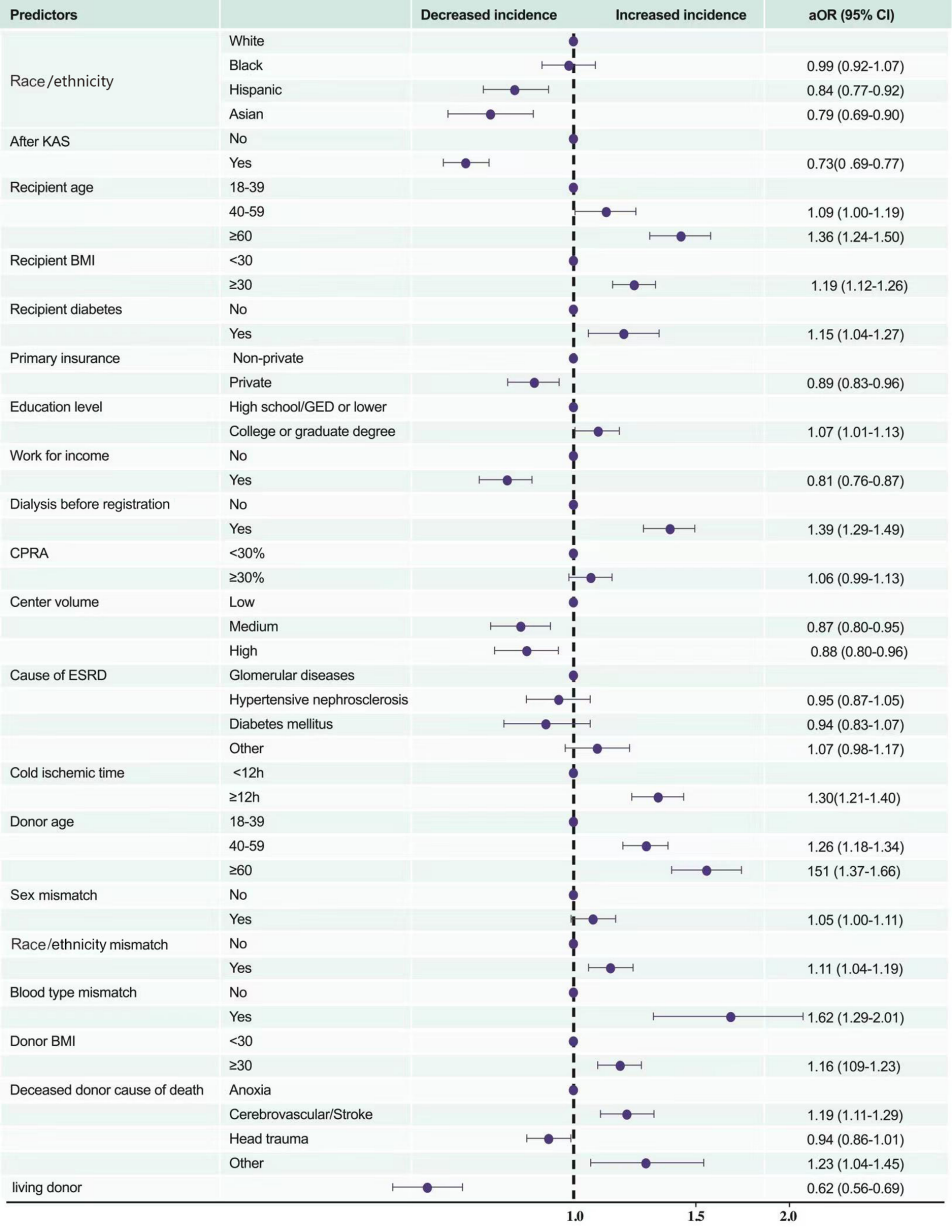
Multivariate predictors of posttransplant in-hospital death or PNF. KAS, Kidney Allocation System BMI, body mass index (calculated as weight in kilograms divided by height in metres squared); CPRA, calculated panel reactive antibody; ESRD, end-stage renal disease; DM, diabetes mellitus; PNF, primary nonfunction.

In univariate logistic regression analysis, compared to white recipients, black recipients (OR [95% CI] 1.29 [1.21–1.38]) had the highest risk of PNF^#^, whereas Hispanic, and Asian patients had similar risks (OR [95% CI] 0.95 [0.88–1.03], and 0.90 [0.79–1.02], respectively). After controlling for recipients' age, BMI, history of diabetes, primary insurance, education level, work state, dialysis time, CPRA, centre volume, the implementation of KAS, and causes of ESRD, donors' age, cold ischaemic time, BMI, cause of death, race/ethnicity mismatch, and sex mismatch between recipients and donors, the risk in black recipients was similar to that of white recipients (aOR 0.99, 95% CI 0.92–1.07), that is, the worst overall posttransplant prognosis. With regard to the recipients' characteristics, most variables had a similar relationship with PNF^#^ as did the characteristics of candidates on the waiting-list death*, although some factors, such as obesity (BMI ≥30) and higher education level (college or graduate degree), had the opposite relationship (associated with higher probability of PNF^#^ aOR [95% CI] 1.19 [1.12–1.26] and 1.07 [1.01–1.13], respectively). Notably, CPRA was not associated with PNF^#^ (1.06 [0.99–1.13]). With regard to donor characteristics, older age (age ≥60 years), obesity, cold ischaemic time ≥12 h, race/ethnicity mismatch, and ABO mismatch were associated with higher probability of PNF^#^ (1.51 [1.37–1.66], 1.16 [1.09–1.23], 1.30 [1.21–1.40], 1.11 [1.04–1.19], and 1.62 [1.29–2.01], respectively; Figure 6).

### Role of KAS

**Figure 7:**
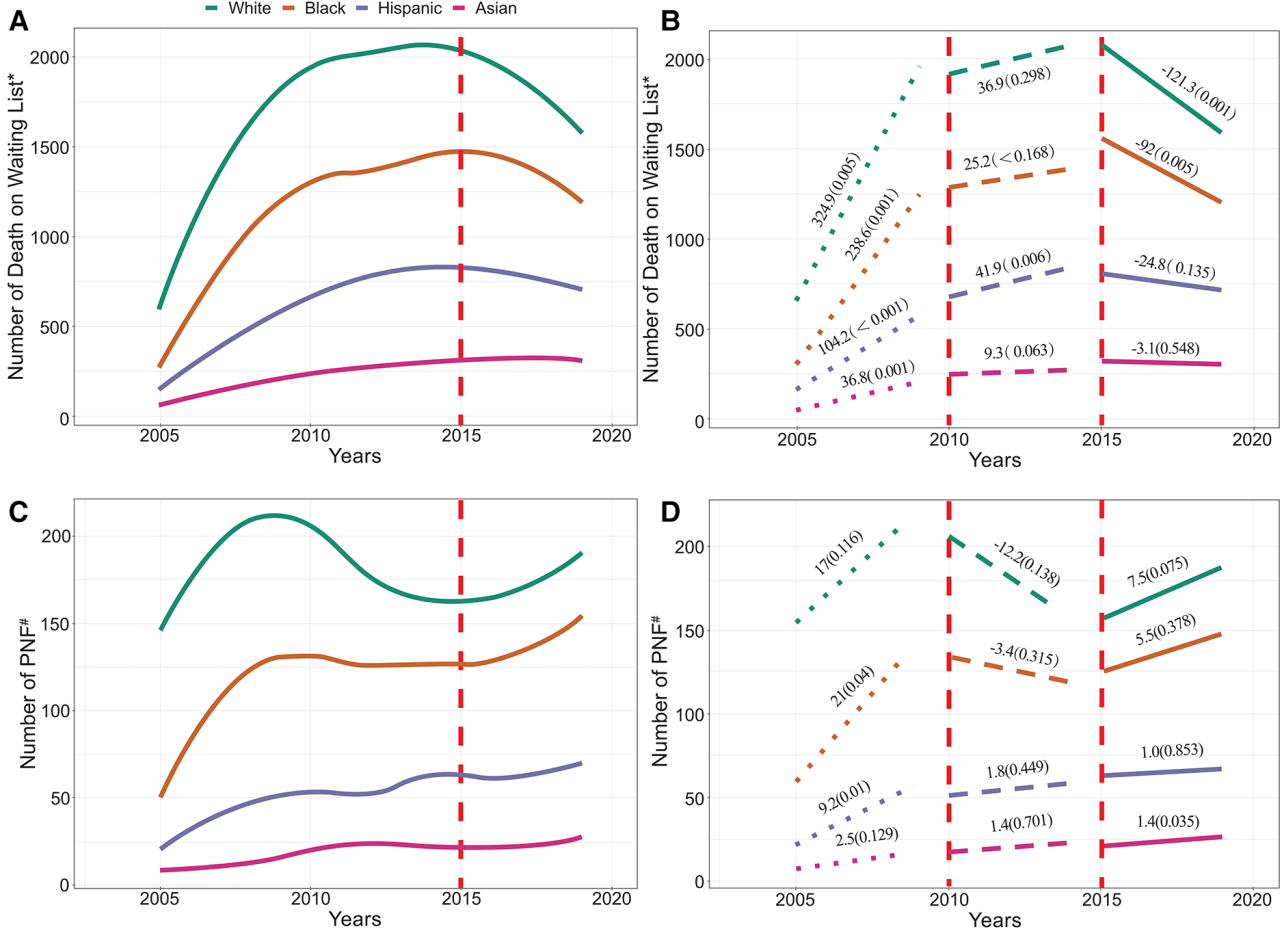
(**A**) illustrates the number of patients who died on the waiting list, and (**B**) depict its trend. (**C**) illustrates the number of recipients with PNF or who died posttransplant, and (**D**) depict its trend. *Includes patients who were removed from the list due to deterioration. #Includes patients who died posttransplant. The vertical line in the figure indicates the start time of KAS.

The number of deaths (including patients who were removed for deterioration) among white, black, Hispanic, and Asian patients increased yearly between 2005 and 2010, but slowed down thereafter until 2015. After the implementation of KAS in December 2014, the number of deaths per year for white, black, Hispanic, and Asian patients on the waiting list decreased (Figure 7B). The number of PNF^#^ in KT recipients increased yearly between 2005 and 2010 in white, black, Hispanic, and Asian patients; and, between 2010 and 2015, was moderate in Hispanic and Asian patients, but showed a downward trend in white and black patients. After the implementation of KAS in December 2014, the number of PNF^#^ in white and black patients showed an upward trend again, which further slowed in Hispanic patients, whereas it maintained the original upward trend in Asian patients (Figure 7D).

The mean KDPI of kidneys allocated to white (0.43 ± 0.27 vs. 0.45 ± 0.26) and Hispanic patients (0.42 ± 0.27 vs. 0.44 ± 0.26) increased significantly after KAS implementation but decreased significantly for black (0.46 ± 0.27 vs. 0.45 ± 0.25) and Asian (0.49 ± 0.27 vs. 0.47 ± 0.27) patients (*P *< 0.001 for all, Table 3).

**Table 3:** KDPI of kidneys allocated to patients of different races/ethnicities before and after KAS implementation.

KDPI	Before KAS	After KAS	*p*
White (mean (SD))	0.43 (0.27)	0.45 (0.26)	<0.001
Black (mean (SD))	0.46 (0.27)	0.45 (0.25)	<0.001
Hispanic (mean (SD))	0.42 (0.27)	0.44 (0.26)	<0.001
Asian (mean (SD))	0.49 (0.27)	0.47 (0.27)	<0.001

KDPI, Kidney Donor Profile Index; KAS, Kidney Allocation System; SD, standard deviation.

### Causes of waiting-list and posttransplant in-hospital deaths

The primary causes of death among these four groups are summarised in Table 4. Deaths due to cardiovascular or cerebrovascular causes (14.2%), infection (4.3%), others/unknown (40.8%), and clinical deterioration (41.1%) were the four major contributors for removal from the waiting list. Death due to other reasons includes miscellaneous causes, cancer, renal failure, haemorrhage, trauma, and other factors. Cardiovascular or cerebrovascular disease is the leading cause of death, and it appears to be a more frequent cause of waiting-list death* among Asians (16.7%) compared with white (13.3%), black (14.0%), or Hispanic (16.0%) patients (*P *< 0.001). Among recipients who died before discharge, cardiovascular or cerebrovascular death (40.3%), infection (16.4%), and other reasons/unknown (43.3%) were the main causes. As the leading cause of death, cardiovascular or cerebrovascular death occurred less frequently among Hispanic (34.4%) than in white (40.6%), black (42.6%), or Asian (40.3%) KT recipients. Infection was a more frequent cause among Asian (19.4%) than in white (19.0%), black (12.5%), or Hispanic (15.2%) KT recipients, but the differences in distribution of cause of death did not reach statistical significance (*P *= 0.202, Table 4).

**Table 4:** Distribution of causes of waiting-list death* and posttransplant in-hospital death.

	White	Black	Hispanic	Asian	Total	*p*
Waiting-List death* (%)						<0.001
Cardiovascular or cerebrovascular	6,246 (13.3)	4,547 (14.0)	2,906 (16.0)	1,087 (16.7)	14,786 (14.2)	
Infection	2,017 (4.3)	1,219 (3.7)	946 (5.2)	267 (4.1)	4,449 (4.3)	
Others/unknown	19,521 (41.5)	13,492 (41.4)	6,846 (37.7)	2,676 (41.1)	42,535 (40.8)	
Removal (deteriorated)	19,275 (41.0)	13,298 (40.8)	7,476 (41.1)	2,479 (38.1)	42,828 (41.1)	
Posttransplant in-hospital death (%)						0.202
Cardiovascular or cerebrovascular	163 (40.6)	113 (42.6)	43 (34.4)	25 (40.3)	344 (40.3)	
Infection	76 (19.0)	33 (12.5)	19 (15.2)	12 (19.4)	140 (16.4)	
Others/unknown	162 (40.4)	119 (44.9)	63 (50.4)	25 (40.3)	369 (43.3)	

* Includes patients who were removed from the list due to deterioration.

## Discussion

We investigated the relationship between race/ethnicity (white, Hispanic, black, and Asian) and the prognosis of candidates and recipients in this study, which included 516,451 patients waiting for KT between July 1, 2004 and March 31, 2020, whose data were recorded in the OPTN database. Furthermore, we explored the relationship between KAS and the prognosis of candidates and recipients. This study had four major findings. First white patients with KT were older, more likely to have private insurance, and less likely to have a history of diabetes. Second, white patients were at the highest risk of dying on the waiting list or becoming too sick for KT, whereas Asian patients had the lowest risk. Third, black recipients had the highest risk of PNF^#^; however, after controlling for some confounding factors, black recipients had a similar risk as white recipients but with a higher risk of PNF^#^ than their Hispanic and Asian counterparts. Finally, the implementation of KAS were associated with lower risk of waiting-list mortality* but were associated with higher risk of PNF^#^ in white and black KT recipients.

Our study found that despite the higher prevalence of comorbidities, barriers to private healthcare, and lower socioeconomic status, racial minorities have a survival advantage compared to white KT candidates. This finding supports previous studies on the effects of racial differences in survival among patients undergoing dialysis (14–19), which showed that black or Hispanic patients survive longer than their white counterparts, indicating a survival paradox (20, 21). Our findings are consistent with the results of these studies, probably because approximately 76.3% of the candidates listed for KT in our study were also on dialysis.

Possible explanations include the higher rates of discontinuation of dialysis therapy, which may account for the lower survival rates of white candidates. Agunbiade et al. (22) found that white patients tended to have an earlier desire to quit dialysis, which may account for their higher mortality. Our findings also indicate that advanced age is a critical risk factor for waiting-list death*, and white candidates were significantly older than candidates of other races/ethnicities (Table 1). Moreover, this survival paradox may partly be subjected to referral bias. Severely ill minority patients with ESRD may be less likely to be offered, elect to initiate dialysis (15), or be referred for KT (4) than their white counterparts. For example, in the United States, the black population accounts for 12%, but represents 36% of the ESRD population. In this analysis, however, they made up only 29% of the OPTN database (23).

In addition to the survival paradox that may be caused by racial differences, the obesity paradox was detected in our study: that is, obesity is a protective factor for waiting-list death*. In the general population, obesity is associated with an increased cardiovascular risk and decreased survival (24). In patients with ESRD, however, an ‘obesity paradox’ has been consistently reported (24–26), namely, a higher BMI is paradoxically associated with better survival (24). The relatively better nutritional status of ESRD patients with high BMI may be responsible for the reduced mortality. Notably, the improved survival associated with obesity was in the adjusted analysis, and that the covariates included diabetes, which is typically associated with obesity in adults.

Given that sicker patients on the KT waiting list may also have a poor perioperative prognosis, we analysed PNF^#^ for additional insights into waiting-list prognosis. We found that black recipients had the highest risk of PNF^#^. However, after controlling for confounding factors, both black and white recipients had the worst early postoperative prognosis compared with their Hispanic and Asian counterparts. This finding is similar to the prognosis of candidates; that is, although white patients are significantly better off than racial minority patients in terms of medical and socioeconomic factors, the early posttransplant prognosis is still the worst. However, unlike the candidates' prognosis, the early posttransplant prognosis of black recipients was significantly worse than that of their white counterparts before adjusting for confounders. Presumably, despite the similar baseline characteristics of recipients and candidates, white patients were allocated better kidneys than black patients. For example, kidneys allocated to racial minority patients tend to have characteristics such as longer ischaemia times and higher racial mismatch rates, which are detrimental to the early posttransplant prognosis of KT recipients.

Our study demonstrates that the implementation of KAS was associated with the number of deaths among candidates on the waiting list. Taber et al. (27) reported that KAS implementation led to significant changes in recipient demographics, increasing the proportion of African Americans, Hispanics, and those with more comorbid conditions. Furthermore, although sensitisation status is associated with higher death (28, 29), KAS has increased the likelihood that highly sensitised recipients (CPRA 99%–100%) would receive transplants (30). Therefore, the proportion of patients on the waiting list with high mortality will be reduced. The results of these studies support our findings.

However, few studies have reported the relationship between KAS implementation and PNF in recipients. Our study found that KAS was associated with increasing number of early posttransplant deaths or PNF in white and black recipients, but not in Hispanic and Asian recipients (Figure 7). However, the reasons for the different relationship between KAS and posttransplant PNF or death in recipients of different race/ethnicity require further investigation.

The strength of this study lies in its large sample size of 516,451 participants. In addition, the study included a diverse racial/ethnic group of patients, including whites, blacks, Hispanics, and Asians. However, this study had some limitations. First, as the database had only limited patient-level information, we could not control for unmeasured confounders, which could be residual confounding factors. For instance, the methods and drugs used to treat terminally ill patients on waiting lists among different races/ethnicities may vary, but data are not available. Second, we were limited in our ability to exclude candidates who were multi-registered, which may have contributed to a potential bias. However, given that this phenomenon occurs across all racial/ethnic groups, it should not have a substantial effect on the results. Third, smaller racial/ethnic groups were excluded as the number of patients was substantially small and heterogeneous to make meaningful conclusions. Fourth, the OPTN database classifies patients as white, black, Hispanic, Asian, American Indian/Alaska Native and others, however, Hispanic/Latino refers to ethnicity only. Thus the “Hispanic” category in the current study may include patients of white race and black race, which means that any conclusions about the Hispanic category are not independent of race differences. Finally, the analysis used United States data and the background provided is United States-centric and does not encompass the broader issues of racial/ethnic intersection in transplantation globally.

In conclusion, race/ethnicity was associated with the prognosis of candidates listed for KT and the early posttransplant prognosis of KT recipients. Despite having better socioeconomic status and allocated better kidneys, white patients have the worst prognosis during the waiting period. Black recipients and white recipients have higher posttransplant in-hospital mortality or PNF. KAS implementation was associated with improved waiting-list survival across all four races/ethnicities but impaired early posttransplant prognosis for white and black recipients.

## Data Availability

Publicly available datasets were analyzed in this study. This data can be found here: https://optn.transplant.hrsa.gov/data/request-data/.
